# Co-delivery of cisplatin and paclitaxel by folic acid conjugated amphiphilic PEG-PLGA copolymer nanoparticles for the treatment of non-small lung cancer

**DOI:** 10.18632/oncotarget.6243

**Published:** 2015-10-26

**Authors:** Zelai He, Jingwen Huang, Yuanyuan Xu, Xiangyu Zhang, Yanwei Teng, Can Huang, Yufeng Wu, Xi Zhang, Huijun Zhang, Wenjie Sun

**Affiliations:** ^1^ Department of Radiation Oncology, Fudan University Shanghai Cancer Center, Department of Oncology, Shanghai Medical College, Fudan University, Shanghai, China; ^2^ The Second Affiliated Hospital and Yuying Children's Hospital of Wenzhou Medical University, Wenzhou, China; ^3^ State Key Laboratory of Oncogenes and Related Genes, Shanghai Cancer Institute, Renji Hospital, School of Medicine, Shanghai Jiao Tong University, Shanghai, China; ^4^ Department of Internal Medicine, Affiliated Cancer Hospital of Zhengzhou University, Henan Cancer Hospital, Zhengzhou, China; ^5^ Department of Cardio-thoracic Surgery, Huashan Hospital, Fudan University, Shanghai, China

**Keywords:** nanoparticles, PLGA, biocompatibility, chemotherapy, cytotoxicity

## Abstract

An amphiphilic copolymer, folic acid (FA) modified poly(ethylene glycol)-poly(lactic-co-glycolic acid) (FA-PEG-PLGA) was prepared and explored as a nanometer carrier for the co-delivery of cisplatin (cis-diaminodichloroplatinum, CDDP) and paclitaxel (PTX). CDDP and PTX were encapsulated inside the hydrophobic inner core and chelated to the middle shell, respectively. PEG provided the outer corona for prolonged circulation. An *in vitro* release profile of the CDDP + PTX-encapsulated nanoparticles revealed that the PTX chelation cross-link prevented an initial burst release of CDDP. After an incubation period of 24 hours, the CDDP+PTX-encapsulated nanoparticles exhibited a highly synergistic effect for the inhibition of A549 (FA receptor negative) and M109 (FA receptor positive) lung cancer cell line proliferation. Pharmacokinetic experiment and distribution research shows that nanoparticles have longer circulation time in the blood and can prolong the treatment times of chemotherapeutic drugs. For the *in vivo* treatment of A549 cells xeno-graft lung tumor, the CDDP+PTX-encapsulated nanoparticles displayed an obvious tumor inhibiting effect with an 89.96% tumor suppression rate (TSR). This TSR was significantly higher than that of free chemotherapy drug combination or nanoparticles with a single drug. For M109 cells xeno-graft tumor, the TSR was 95.03%. *In vitro* and *in vivo* experiments have all shown that the CDDP+PTX-encapsulated nanoparticles have better targeting and antitumor effects in M109 cells than CDDP+PTX-loaded PEG-PLGA nanoparticles (*p* < 0.05). In addition, more importantly, the enhanced anti-tumor efficacy of the CDDP+PTX-encapsulated nanoparticles came with reduced side-effects. No obvious body weight loss or functional changes occurred within blood components, liver, or kidneys during the treatment of A549 and M109 tumor-bearing mice with the CDDP+PTX-encapsulated nanoparticles. Thus, the FA modified amphiphilic copolymer-based combination of CDDP and PTX may provide useful guidance for effective and safe cancer chemotherapy, especially in tumors with high FA receptor expression.

## INTRODUCTION

In small-molecule-based chemotherapy, the use of a single agent often cannot achieve complete tumor remission due to the rapid development of drug resistance of tumor cells. However, the combination therapy of multiple drugs with different action mechanisms, has proven to be an effective strategy in clinical cancer treatments. Combination chemotherapy offers several benefits. First, applying multiple drugs with different action mechanisms can delay the related cancer cell mutations and the cancer adaption process. Second, multiple drugs targeting the tumor cells can function synergistically, thereby, reducing drug side effects by decreasing drug doses and achieving synergistic therapeutic efficacy. [1, 2]

However, it is difficult to combine free drugs to obtain optimal anticancer effect. The combination of free drugs often brought more serious toxic side effects to humans, which has been a serious problem in clinical cancer treatments. In addition, their different biochemical properties and pharmacokinetic characteristics affect synergistic therapeutic efficacy. [3] The combination of cisplatin (cis-diaminodichloroplatinum, CDDP) and paclitaxel (PTX) is a prime example of this phenomenon. CDDP is one of the most widely used DNA-modifying chemotheraputic drugs which can induce each cell cycle of cancer cell to apoptosis. [4] PTX, as a representative microtubule-stabilizing chemotherapeutic drug, is highly hydrophobic with very poor water solubility. The combination of free-CDDP and free-PTX has shown a positive synergistic effect against a wide range of tumors due to different action mechanisms of CDDP and PTX. Currently, the combination of free-CDDP and free-PTX drugs has become the first-line chemotherapeutic agent for advanced breast cancer, advanced non-small-cell lung cancer (NSCLC), advanced gastric cancer, ovarian cancer, etc. However, drug combination is not as simple as just putting things together. One of the most important dose-limiting side effects of CDDP is nephrotoxicity. This increased side effect was observed when CDDP was co-administered with PTX for treatment of lung cancer, in comparison to administering CDDP alone. In the advanced transitional urothelium carcinoma study, the combination of free-CDDP and free-PTX also showed an effective clinical response. However, there was also an increased toxic effect. Therefore, it could not be applied to patients with poor performance status or those over 70 years of age. In the advanced ovarian cancer study, the combination of free-CDDP and free-PTX did not significantly improve the survival times. However, it did increase hematological toxicities, nephrotoxicity, and hospitalizations. Therefore, the additional clinical benefit gained from the combination chemotherapy of free-CDDP and free-PTX was discounted because of the increased side effects.

Advances in nanotechnology have provided us with an unprecedented opportunity for novel combination strategy and drug targeting delivery. [5] Compared to the combination of free drugs, the combination of several kinds of drugs, within a single nanocarrier, can remarkably reduce non-specific interactions of free drugs with normal tissues. In addition, the drug combination can increase the accumulation of combination drugs in solid tumors through the enhanced permeability and retention effect (EPR). Therefore, this treatment method enhances the chemotherapeutic efficacy while decreasing toxicity and other side effects. [6, 7] Furthermore, such co-delivery systems guarantee the simultaneous delivery of sufficient amount of drugs to tumor site while giving full play to the synergistic effect and the improved antitumor efficacy.

However, for CDDP and PTX, the challenge of entrapment of the two drugs into a co-delivery system might primarily be due to the hydrophilic, metal complex nature of CDDP and the hydrophobic nature of PTX. Therefore, although the combination of free-CDDP and free-PTX has been used as the first-line treatment for various solid tumors, the co-delivery of CDDP and PTX was rarely investigated. Song et al. employed methoxy- poly(ethylene glycol)-b-poly(L-glutamic acid)-b-poly(L-phenylalanine) (mPEG-b-P(Glu)-b-P(Phe)) triblock copolymer nanoparticles (NPs) as carriers to co-delivery CDDP and PTX, and received a synergistic antitumor effect *in vitro*/*in vivo*. [8] However, such NPs lack a targeting group which leads to lack of active targeting and reduces the targeting efficiency.

In order to co-deliver multiple chemotherapeutic agents with strong polymer/agent interactions and robust construct stability, an amphiphilic copolymer, folic acid (FA) modified poly(ethylene glycol)-poly(lactic-co-glycolic acid) (FA-PEG-PLGA), was used as a favorable carrier for the co-delivery of CDDP and PTX in the study. In the NPs, covalent conjugation of FA could provide active targeting and enhanced specificity uptake of the NPs by overexpression of folate receptors by tumor cells while sparing in normal tissues, thus, improving drug efficacy and reducing its side effects. [9] Poly(ethylene glycol) (PEG) was the outer corona and provides the prolonged blood circulation of the NPs by reducing non-specific interactions with blood components. The PLGA component of hydrophobic serves as a reservoir for the lipophilic drug. The anionic copolymer provides the strong electrostatic interaction with cationic CDDP. [10]

The physiochemical properties, stability, *in vitro* drug release behavior, pharmacokinetic characteristics, and *in vivo* biodistribution were investigated. The synergistic antitumor effect of the CDDP and PTX co-delivered FA-PEG-PLGA NPs (Co-FA-NPs) was evaluated both *in vitro* and *in vivo*.

## RESULTS AND DISCUSSION

### Characterizations of FA-PEG-PLGA polymer

**Figure 1:**
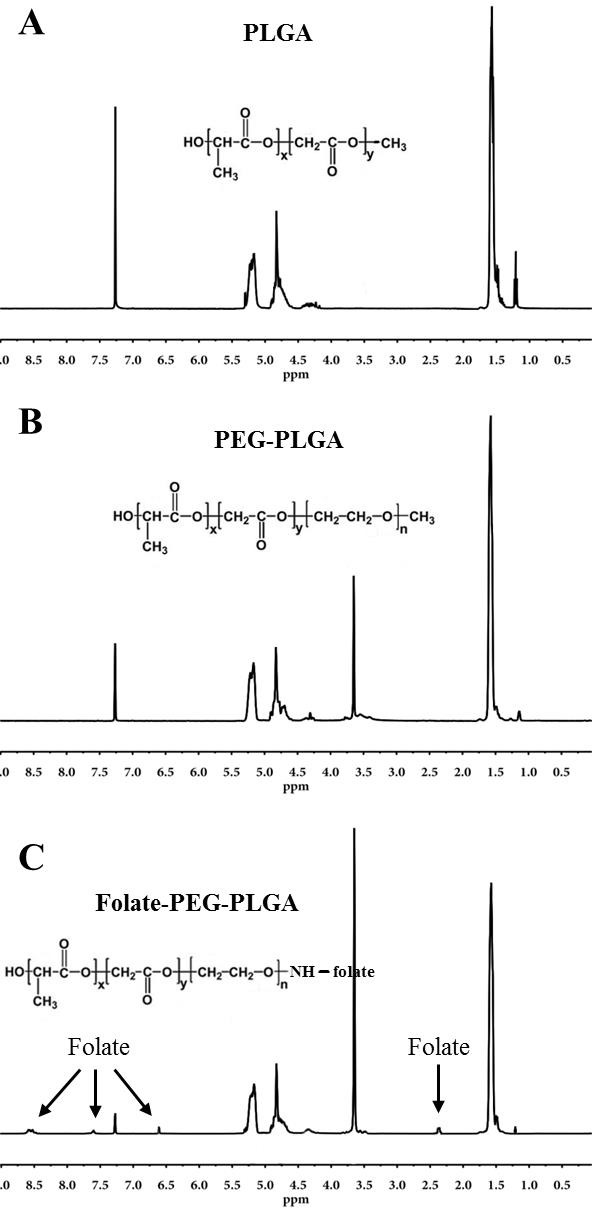
Characterization of the amphililic copolymers ^1^H-NMR spectrum of PLGA **A.**, PEG-PLGA **B.**, and FA-PEG-PLGA **C.** copolymer.

The obtained PLGA, PEG-PLGA, and FA-PEG-PLGA polymer was characterized by ^1^H-NMR. In ^1^H-NMR of PLGA (Figure 1A), PEG-PLGA (Figure 1B) and FA-PEG-PLGA (Figure 1C), the integrated signals, around 1.6 ppm, were attributed to the methyl protons of the _L_- and _D_-lactic acid units. The large peak, at 3.6 ppm, in Figure 1B was assigned to the methylene protons of the PEG. The peaks of the glycolic acid CH_2_ protons were at 4.8 ppm and the lactic acid CH were assigned at 5.2 ppm. The resulting conjugates of FA were confirmed by ^1^H-NMR, and the peak of FA was assigned at 2.3 ppm (γ-CH_2_, glutamic acid), 6.6 and 7.6 ppm (aromatic protons), and 8.6 ppm (pteridine proton) (Figure 1C). [32] The aforementioned is indicative of the successful synthesis of PLGA, PEG-PLGA and FA-PEG-PLGA copolymer. The final conjugation percentage of folic acid to PEG-PLGA was 43.1%.

### Size, zeta potential, morphology, and drug loading of NPs

FA-PEG-PLGA, as amphiphilic copolymers, can form emulsion in water solution and finally obtain the NPs of shell-core structure (supplementary Scheme 1). The NPs have appropriate size to evade endocytosis and destruction by the reticuloendothelial system (RES). Therefore, they are able to survive longer in circulation. In addition, the appropriate size may bring to ideal targeting. The obtained NPs were characterized by DLS. Table 1 summarized the average size of these NPs. The average size and zeta potential of blank NPs was 134.71±6.42 nm and - 26.12±1.23 mV, respectively. The size polydispersity index was 0.165. CDDP-FA-NPs and PTX-FA-NPs were larger than blank NPs, which indicates that CDDP and PTX in the core of the NPs increased the volume. Co-delivery NPs showed the larger diameter and smaller polydispersity than blank, CDDP-, and PTX-FA-NPs, which could be attributed to presence of hydrophobic PTX, thus, making the amphiphilic polymers form an intensive structure. [2, 33, 34] In co-delivery NPs, the size was increased with increasing PTX content (partial data not shown). In addition, the PTX-FA-NPs were also larger than CDDP-FA-NPs. These indicated that PTX of hydrophobic has more effects for size than CDDP of polarity. When comparing CDDP-FA-NPs and PTX-FA-NPs with blank NPs, blank NPs have the lowest zeta potential. In co-delivery NPs, the zeta potential was increased with increase of CDDP content ratio. These indicated that CDDP of large polarity has more effects for zeta potential than PTX of non-polarity.

**Table 1:** Characterization of blank NPs and drug encapsulated NPs

Groups	Particle size (nm)	Polydispersity	Zeta potential (mV)
FA-PEG-PLGA NPs	134.71±6.42	0.165±0.015	−26.12±1.23
CDDP-FA-NPs	149.38±9.04	0.187±0.018	−22.48±0.97
PTX-FA-NPs	161.81±6.97	0.172±0.025	−23.51±1.34
Co-FA-NPs (CDDP: PTX = 2:1)	171.36±8.67	0.133±0.008	−21.50±0.88
Co-FA-NPs (CDDP: PTX = 1:1)	177.23±10.79	0.151±0.013	−22.78±1.16
Co-FA-NPs (CDDP: PTX = 1:2)	185.94±7.43	0.158±0.012	−24.68±1.21
Co-NPs (CDDP: PTX = 1:2)	176.56±4.83	0.161±0.016	−23.07±0.98

Diameter and zeta potential were determined by DLS. Three batches of samples (1 mg/mL) were prepared by directly dissolving NPs in distilled water, then sonication 5 min.

**Figure 2:**
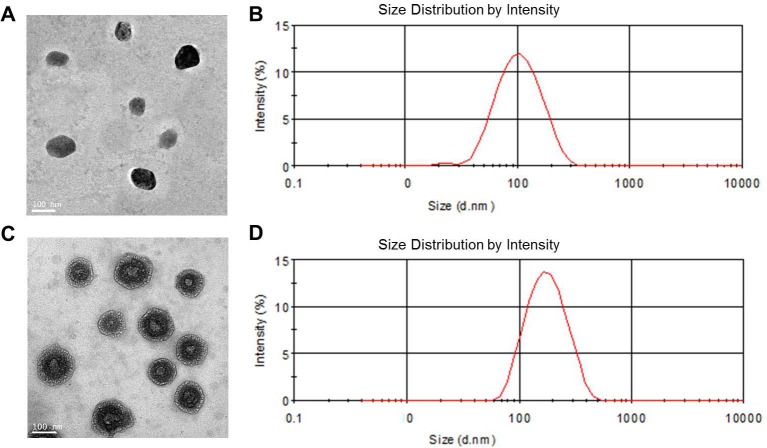
TEM and DLS characterization of NPs **A.** TEM image and **B.** DLS graph of blank NPs; **C.** TEM image and **D.** DLS graph of Co-FA-NPs.

The morphology of blank NPs and Co-FA-NPs was characterized using TEM (Figure 2A). The TEM results show that blank NPs and Co-FA-NPs were single uniformity spherical particles, and the diameter of blank NPs was smaller than the diameter of Co-FA-NPs with the uniformity diameter of 150 nm and an intensive shell-core structure. Meantime, the narrow polydispersity, at a diameter of 180 nm, of Co-FA-NPs was obtained by DLS. The polydispersity was narrower than 0.165 of blank NPs and the diameter were larger than 134.71 nm of blank NPs (Figure 2B).

As shown in Table 2, when the polymer/drug ratio was 40:2:1, CDDP and PTX in Co-FA-NPs have the highest loaded efficiencies at 62.43 and 71.71%, respectively. The highest DLC was 12.07% at a polymer/drug ratio of 40:2:4.

**Table 2:** Different polymer/drug ratio impact on DLE and DLC of drug-loaded NPs

Nanoparticles	Polymer:CDDP:PTX ratio in Co-FA-NPs			CDDP-FA-NPs	PTX-FA-NPs	Co-NPs (CDDP:PTX=1:2)
	40:2:1	40:2:2	40:2:4			
DLE of CDDP (%)	62.43±1.36	59.84±0.71	49.67±0.98	76.92±1.11	-	51.24±1.78
DLE of PTX (%)	71.71±1.08	66.35±1.83	58.46±0.38	-	79.49±0.76	59.73±0.94
DLC (%)	7.58±0.29	11.09±0.41	12.07±0.21	4.68±0.07	5.83±0.04	12.41±1.42

### Stability and *in vitro* drug release of co-delivery NPs

**Figure 3:**
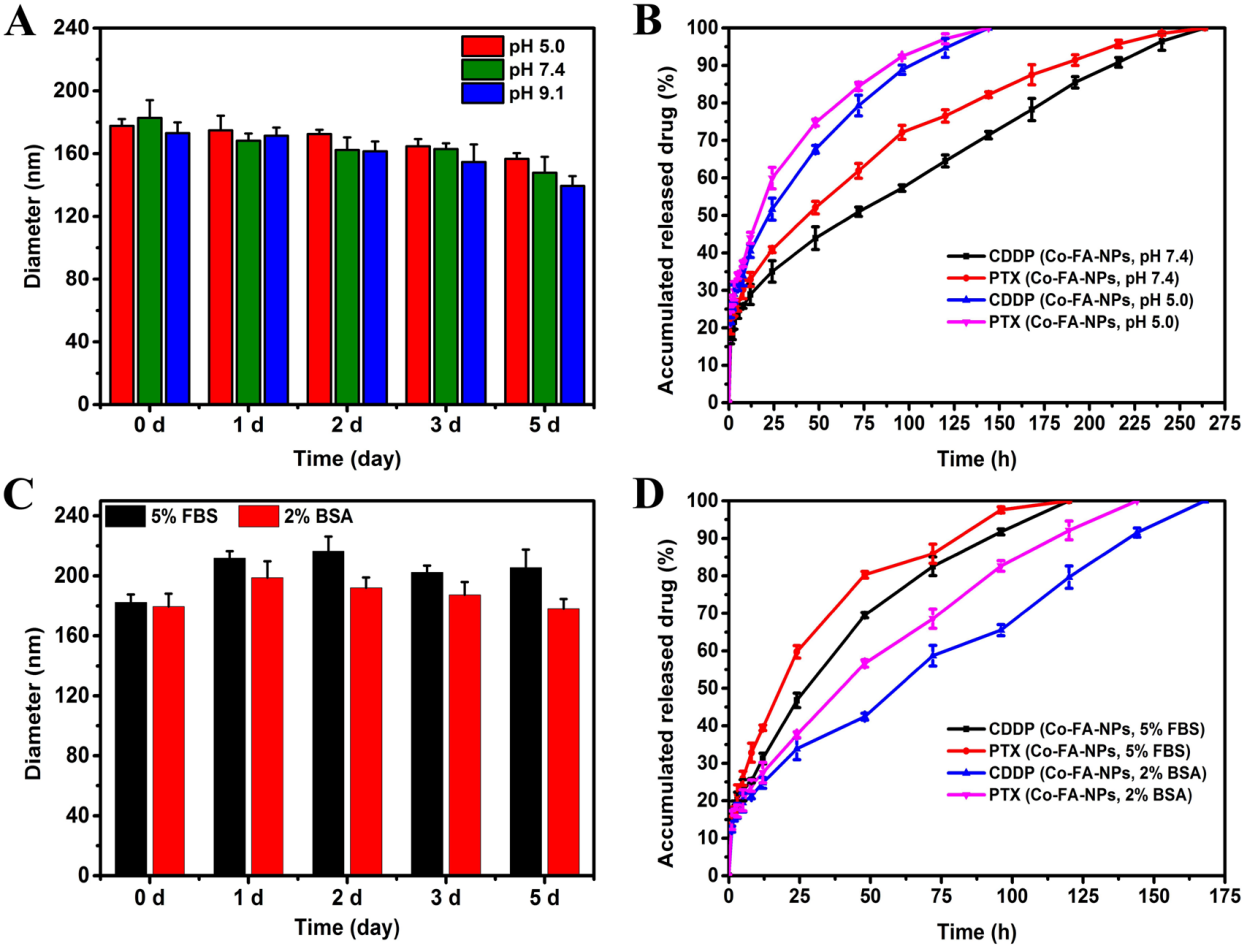
Stability profiles **A.** and release profiles **B.** of CDDP and PTX from Co-FA-NPs in PBS at 37°C. Stability profiles **C.** and release profiles **D.** of CDDP and PTX from Co-FA-NPs in 5% FBS or 2% BSA at 37°C. **A.** Stability of Co-FA-NPs in solutions with varying pH as judged by their diameter. **B.** Release profiles of CDDP and PTX from Co-FA-NPs in solutions of pH 5.0 and pH 7.4. **C.** Stability of Co-FA-NPs in solutions with 5% FBS or 2% BSA as judged by their diameter. **D.** Release profiles of CDDP and PTX from Co-FA-NPs in solutions of 5% FBS or 2% BSA.

Clinical pharmaceutical applications of NPs formulation should have ideal stability because the stability of NPs is crucial for *in vitro* long-term storage and transportation, *in vivo* targeting, and long circulation. [2] A total of 1 mg/mL of NPs were prepared by directly dissolving co-delivery NPs (CDDP: PTX = 1:2) in PBS (pH 5.0, 7.4, 9.1), 5% FBS or 2% BSA, then sonicated for 5 min. The NPs were placed at 37°C, and size change was detected. It can be seen, from Figure 3A, there was no significant change in the size of Co-FA-NPs (CDDP: PTX = 1:2) within 5 days, which was beneficial for clinical applications. However, compared to the acidic media (pH 5.0), a slightly smaller size was still observed in alkaline media (pH 9.1). It can determined that poly(lactic acid) polymer was faster to degrade in alkaline media than that in acidic media. However, the degradation did not obviously change drug release within 5 days. It is possible that the pH value effect on drug release was greater than that of NPs degradation (Figure 3B).

**Figure 4:**
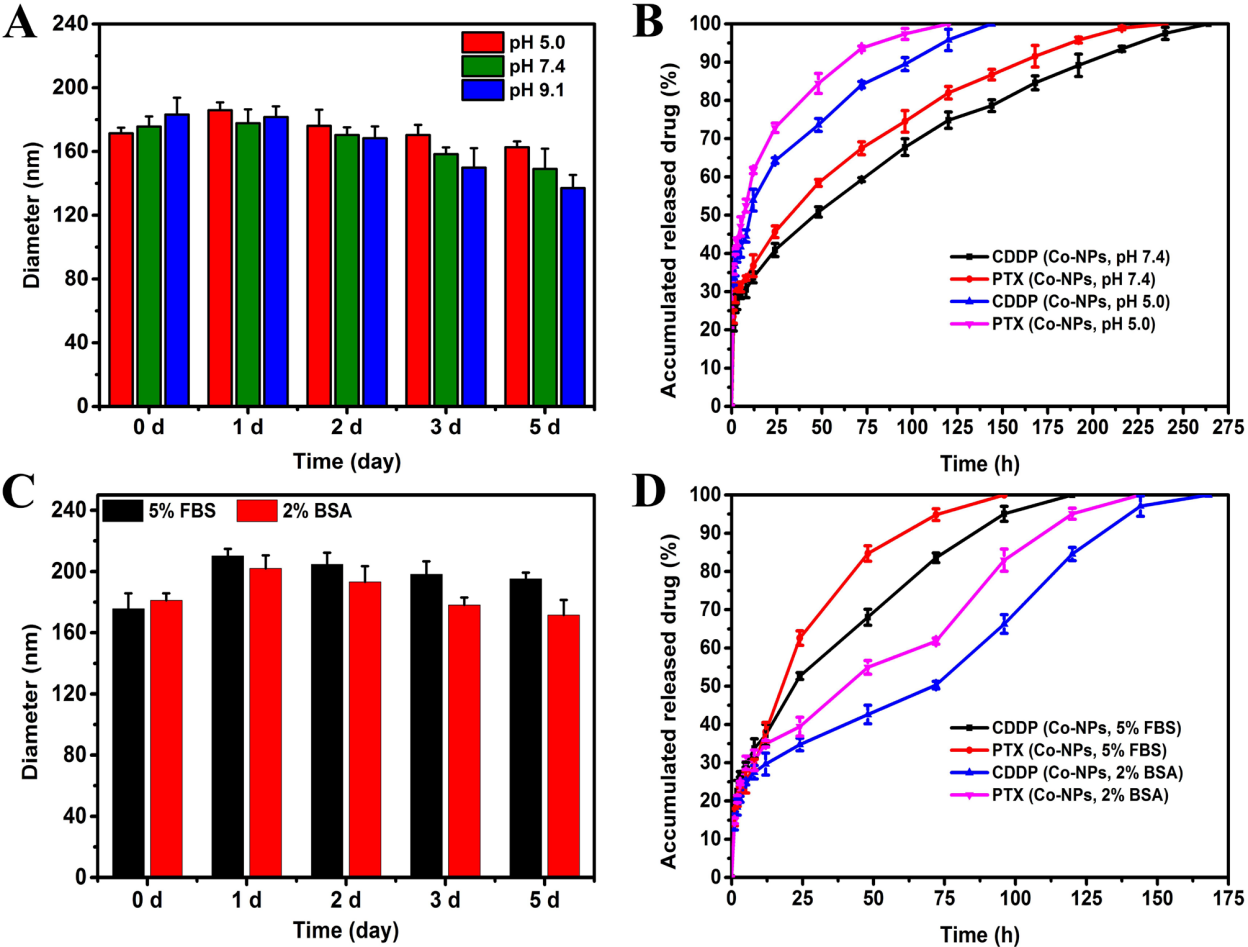
Stability profiles **A.** and release profiles **B.** of CDDP and PTX from Co-NPs in PBS at 37°C. Stability profiles **C.** and release profiles **D.** of CDDP and PTX from Co-NPs in 5% FBS or 2% BSA at 37°C. **A.** Stability of Co-NPs in solutions with varying pH as judged by their diameter. **B.** Release profiles of CDDP and PTX from Co-NPs in solutions of pH 5.0 and pH 7.4. **C.** Stability of Co-NPs in solutions with 5% FBS or 2% BSA as judged by their diameter. **D.** Release profiles of CDDP and PTX from Co-NPs in solutions of 5% FBS or 2% BSA.

A distinct advantage offered by co-delivery systems of multiple drugs is controlled release of the drug, which greatly intensifies the synergistic bioavailability of the drugs and lessens the resultant side effects of these drugs to healthy body tissues by the EPR effects. [35] *In vitro* release profiles of Co-FA-NPs were acquired in PBS with a pH of 5.0 and an additional solution with a pH of 7.4. The release processes of both CDDP and PTX were affected by a change in the pH value of the solution (Figure 3B). The associated releases of CDDP and PTX, in PBS solution with a pH of 7.4 (blood environment), were slow and requires approximately 264 hours to completely release. According to the study, the Co-FA-NPs were primarily binding to FA receptors in the cell membrane, taken up by cells via endocytosis or potential pinocytosis, then degraded, metabolized, and released the drug in the lysosome (acidic environment). [2, 10, 36] Moreover, the inner environment in the tumor was also acidic (pH 4-5). Consequently, examination of these release profiles of Co-FA-NPs under acidic pH value and blood pH value environment is crucial. In comparison to the release profiles when the pH is 5.0, the release of the drug in pH 7.4 PBS was much slower than that in the acidic environment. For the simulated blood environment, CDDP and PTX were completely released within 150 hours. After co-incubation with 5% FBS or 2% BSA solution, it can been seen that the diameter of Co-FA-NPs were slightly increased within 5 days, however, the increment value were less than 40 and 20 nm, respectively (Figure 3C). The release profiles of CDDP and PTX in FBS and BSA solution were also slow. The release time in 5% FBS was 120 hours, the release time of CDDP and PTX in 2% BSA were 168 and 144 hours, respectively (Figure 3D). Consequently, the release profiles, acquired from CDDP and PTX, indicate the co-delivery system successfully provided the same biochemical and pharmacokinetic characteristics and their synergistic effect. In theory, PTX is initially released once the outermost copolymer layer breaks off. Once the outermost copolymer layer breaks off, the unstable core structure with hydrophobic surface rapidly disintegrates and initiates the release of CDDP. [2, 10] The Co-NPs (CDDP: PTX = 1:2) have the similar stability and release profiles with Co-FA-NPs (CDDP: PTX = 1:2) (Figure 3 and Figure 4).

### *In vitro* cytotoxicity

**Figure 5:**
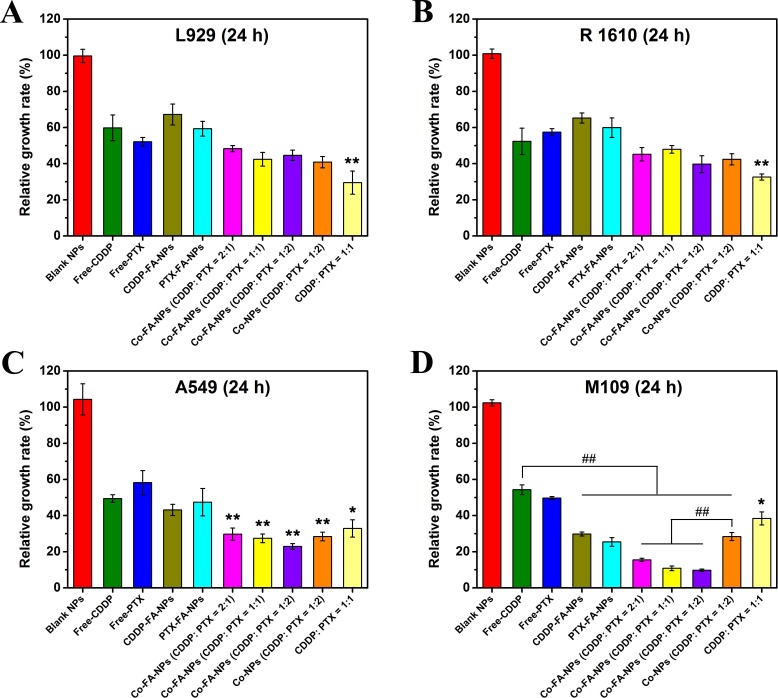
CCK8 assay Cell viability of L929, R 1610, A549, and M109 cells after exposure to free-CDDP, free-PTX, CDDP-FA-NPs, PTX-FA-NPs, free-CDDP + free-PTX, Co-NPs (CDDP: PTX = 1:2), and Co-FA-NPs with different ratio at 37° for 24 hours. The total drug contents in all groups were kept at 10.0 *μ*g/mL for all tests, *p < 0.05 and **p < 0.01 compared with free-CDDP group. ## indicated that the *p* < 0.01.

In our study, the combination of CDDP and PTX was incorporated in order to demonstrate the synergistic effect of multiple chemotherapeutic drugs. L929, R1610, A549, and M109 cells were incubated independently among 96-well plates. Once the cells adhered, they were subsequently treated for a period of 24 hours with the free-CDDP, free-PTX, CDDP-FA-NPs, PTX-FA-NPs, free-CDDP + free-PTX, Co-FA-NPs, and Co-NPs containing 10.0 *μ*g/mL of drug concentration. The blank NPs and without NPs treat-cell were used as controls. Figure 5 clearly represents the significant reduction in tumor cell viability associated with the co-delivery of CDDP and PTX (*p* < 0.01 compared with free-CDDP groups) in A549 and M109 cell lines. For L929, R 1610, and A549 cell lines, free single drug and single drug-loaded NPs were associated with similar cytotoxicity, which confirms that the manner in which the drug is released from the endosomes/lysosomes into the cytosols is highly efficient. However, in L929 and R 1610 cells, the co-delivery NPs exhibited less cytotoxicity which could be due to the fact that the L929 and R1610 cells belong to normal cells which proliferate slower than tumor cells. Doubling time was obviously longer than tumor cells, therefore, the synergistic effect of two chemotherapeutic drugs in the same vehicle was unapparent within 24 hours. In the M109 cell line, the cell viability of single drug-loaded NPs groups was significantly less than that of free-drug groups, and the cell viability of Co-FA-NPs groups was obviously lower than that of Co-NPs (CDDP: PTX = 1:2) (*p* < 0.01). In addition, the cell viability of Co-FA-NPs in M109 cells was lower than that in A549 cells (*p* < 0.05). These should be the covalent conjugations of FA groups which provide active targeting and enhanced uptake of the NPs by M109 cells by overexpressing folate receptors, increasing tumor cell death, and improving drug efficacy.

We also examined the role of drug ratio in the inhibition of cancer cells. In A549 and M109 cells, the viability of co-delivery NPs, with the CDDP/PTX concentration of 1:2, was approximately cut in half in comparison to free-CDDP. Moreover, the co-delivery NPs, with CDDP/PTX concentration of 1:2, exhibited the greatest anti-tumor activity among the two different varieties of lung cancer cells.

The resultant synergistic effect can be obtained through the combination of the associated anti-tumor mechanisms of each drug. It was well-known that CDDP, inserted into DNA, triggers many molecular events that induce apoptosis in cancer cells. PTX has the ability to inhibit microtubule disassembly and produce a highly stabilizing microtubule. This inhibition disrupts the normal metabolism for mitosis and cell proliferation, thus, inducing cell apoptosis. According to our *in vitro* studies, when using CDDP and PTX together with a ratio of 1:2, the synergistic effects could promote and accelerate tumor cell death. The combination of these two individual drugs in any other (2:1 or 1:1) produces lessened anti-tumor efficacy.

### Pharmacokinetic assay

The pharmacokinetics associated with both free-drug and drug-loaded NPs were investigated by tail intravenous injection. Small amounts of the blood was collected from the rats and CDDP, PTX levels were calculated through the use of HPLC. A two-compartment model was the preferred method to assess the many pharmacokinetic parameters of CDDP and PTX in plasma. The pharmacokinetic parameters, which describe the bodily effects from free-drug or drug-loaded NPs, are of extreme importance to the scientific community. The parameters of greatest importance are AUC, CL, MRT, t_1/2_ (half-life) and V_d,ss_. [37, 38, 39] In addition to these parameters describing the behavior of free-drug and drug-loaded NPs, these parameters are also useful for determining future clinical applications.

**Figure 6:**
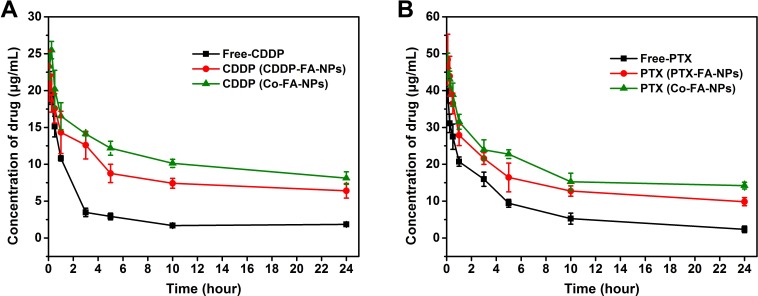
The mean concentration-time profile of CDDP

The pharmacokinetic effects for free-drug and drug-loaded NPs are clearly depicted in Figure 6. Free-CDDP and free-PTX revealed rapid system clearance after administration. Following the CDDP-loaded NPs and PTX-loaded NPs, the Co-FA-NPs was the slowest. The concentration of free-CDDP and free-PTX decreases to less than 3 and 9.5 *μ*g/mL, respectively, within 5 hours. CDDP and PTX concentration, in Co-FA-NPs, remain at approximately 8.13 and 14.22 *μ*g/mL, respectively, up to 24 hours. The Co-FA-NPs shows prolonged blood circulation time. The prolonged blood circulation time is a direct result of the enhanced retention effect of the poly(ethylene glycol) shell that contains a slightly negative surface charge of NPs which is associated with repulsion of negatively charged RBC's.

**A.** and PTX **B.** in plasma after administration of a single dose of CDDP (5 mg/kg), PTX (12 mg/kg), single drug-loaded NPs or Co-FA-NPs (equivalent to CDDP and PTX) to rats. Each point represents the mean ± SD.

The pharmacokinetic parameters are clearly depicted in Table 3. CDDP-loaded NPs treated rats showed a 2.9 fold greater maximum platinum concentration and Co-FA-NPs produced 3.19 times greater in comparison to rats treated with free-CDDP (AUC). MRT for CDDP-loaded NPs is around 1.8 times and Co-FA-NPs is around 2.1 times greater in comparison to free-CDDP. The greater AUC and MRT values for CDDP-loaded NPs and Co-FA-NPs, as compared to free-CDDP, are associated with greater bioavailability of these formulations for systematic circulation. The t_1/2_ of CDDP-loaded NPs is 1.75 times and Co-FA-NPs is 2.08 times greater in comparison to free-CDDP. The V_d,ss_ of CDDP-loaded NPs and Co-FA-NPs is also slightly higher than free-CDDP. The bodily clearance of free-CDDP of the rats is 2.46 times greater in comparison to CDDP-loaded NPs and 2.65 times greater in comparison to Co-FA-NPs. The rule is similar for PTX. AUC, MRT, and t_1/2_ for Co-FA-NPs is, respectively, 1.61, 1.55 and 1.60 times greater in comparison to PTX-loaded NPs while CL is 1.48 times less in comparison to PTX-loaded NPs. These pharmacokinetic parameters predict that CDDP-loaded NPs, PTX-loaded NPs, and Co-FA-NPs promoted stability and prolonged circulation of CDDP and PTX. Decreased stability and circulation time of drug-loaded NPs, in comparison to Co-FA-NPs, is directly related to the faster *in vitro* release rate.

**Table 3:** Pharmacokinetic parameters in mice after administration of free drug, single drug-loaded NPs, and Co-FANPs containing CDDP and/or PTX (mean ± SD, *n* = 6)

	Drug	Dose (mg/kg)	AUC (μg*h/mL)	CL (L/h/kg)	MRT (h)	t_1/2_(h)	V_d,ss_ (L/kg)
Free-drug loaded	Free-CDDP	5	10.09±1.06	0.69±0.03	6.76±1.15	4.91±0.28	2.08±0.07
	Free-PTX	12	14.38±2.67	0.84±0.04	1.17±0.14	0.97±0.09	0.56±0.06
FA-NPs loaded	CDDP	5	29.02±3.41	0.28±0.03	11.85±1.62	8.6±1.14	2.57±0.34
	PTX	12	32.59±3.85	0.49±0.03	1.87±0.23	1.11±0.12	0.57±0.05
Co-FA-NPs	CDDP	5	32.21±2.44	0.26±0.07	14.07±1.46	10.25±1.91	2.74±0.45
	PTX	12	52.34±5.36	0.33±0.02	2.89±0.52	1.78±0.16	0.61±0.11

### iodistribution study *in vivo*

**Figure 7:**
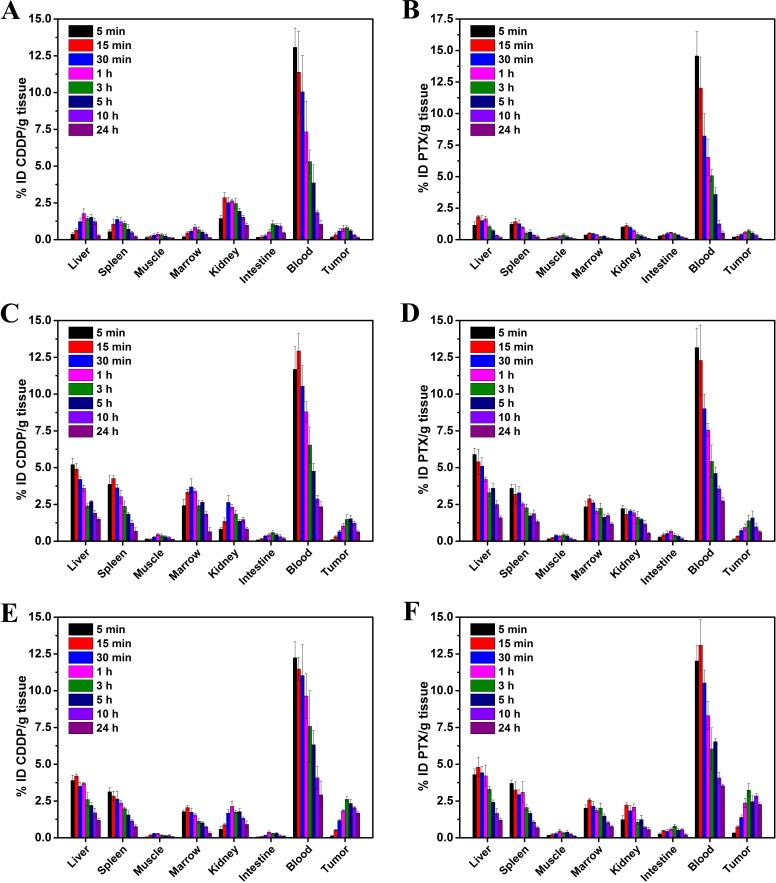
Tissue distribution studies of drug/NPs Tissue distribution of free-CDDP **A.**, free-PTX **B.**, the CDDP in Co-NPs **C.**, the PTX in Co-NPs **D.**, the CDDP in Co-FA-NPs **E.** and the PTX in Co-FA-NPs **F.** at time points of 5, 15, 30 min, 1, 3, 5, 10 and 24 h after intravenous injection of corresponding drug/NPs.

The biodistribution of CDDP and PTX, up to 24 hours (5, 15 and 30 min, 1, 3, 5, 10 and 24 h) following administration in Co-FA-NPs, is shown in Figure 7. According to Figure 7, the concentration of CDDP and PTX, in most organs, decreased over the 24 hour period. The destabilization of co-delivery NPs in the blood compartment initiated the release of CDDP and PTX in the bloodstream. In Co-NPs, the drug was then rapidly cleared through the kidneys where a relatively high level of CDDP was detected (0.82 to 2.64% ID/g), and the PTX was 0.54 to 2.22% ID/g. For Co-FA-NPs, the relatively level of CDDP was 0.58 to 2.23% ID/g, and the PTX was 0.56 to 2.14% ID/g. This was especially true when free-CDDP was measured within the first 15 min post-injection (2.86±0.34% ID/g), and the free-PTX was 1.13±0.14% ID/g. In addition, there were relatively high amounts of CDDP and PTX in Co-FA-NPs measured in the liver (1.2 to 4.2% ID/g) and (1.3 to 4.8% ID/g), respectively. When CDDP and PTX was measured in the spleen, the results were (0.77 to 3.13% ID/g) and (0.69 to 3.7% ID/g), respectively. This is expected because NPs typically accumulate in organs of the mononuclear phagocyte system (MPS). Moreover, at 24 h, CDDP (2.34% ID/g), PTX (2.78% ID/g) in Co-NPs and CDDP (2.92% ID/g), PTX (3.54% ID/g) in Co-FA-NPs within the blood were significantly higher than free-CDDP (1.05% ID/g) and free-PTX (0.53% ID/g) (*p* < 0.01). It indicated that the NPs have good stability and long circulation properties.

In the M109 tumor tissues, at 5 h, CDDP (2.34% ID/g) and PTX (2.46% ID/g) in Co-FA-NPs were significantly higher than Co-NPs (CDDP: 1.52% ID/g; PTX: 1.67% ID/g) (*p* < 0.05). In addition, Co-NPs were significantly higher than free-CDDP (0.61% ID/g) and free-PTX (0.53% ID/g) (*p* < 0.01). Up to 24 h, the differences were further enlarged, CDDP (1.68% ID/g) and PTX (2.27% ID/g) in the Co-FA-NPs groups were significantly higher than Co-NPs (CDDP: 0.63% ID/g; PTX: 0.84% ID/g) (*p* < 0.01). Co-NPs were also significantly higher than free-CDDP (0.14% ID/g) and free-PTX (0.11% ID/g) (*p* < 0.01).

The T/NT (tumor/muscle) was similarly distributed. At 5 h, the T/NT (CDDP: 16.71; PTX: 6.67) in Co-FA-NPs was obviously higher than Co-NPs (CDDP: 4.90; PTX: 4.39). Co-NPs were significantly higher than free-CDDP (2.44) and free-PTX (2.21) (*p* < 0.05). Up to 24 h, the differences were further enlarged, and the T/NT (CDDP: 24; PTX: 18.92) in Co-FA-NPs was significantly higher than Co-NPs (CDDP: 4.85; PTX: 6.4) (*p* < 0.01). In addition, Co-NPs were also significantly higher than free-CDDP (1.27) and free-PTX (1.38) (*p* < 0.01).

These demonstrated that the Co-FA-NPs have better targeting for M109 cell grafted tumors than Co-NPs and free drugs. It may be related to the specific binding of ligand and receptor in the M109 cells surface.

Free-CDDP are mainly distributed in the blood, kidneys, liver, and spleen, with secondary distribution in the small intestine, bone marrow, and tumor. Free-PTX is primarily dispersed in the blood, liver and spleen, with secondary distribution in the kidneys, tumor, and small intestine. The CDDP and PTX in Co-NPs are mainly dispersed to the blood, liver, and spleen, and bone marrow, followed by secondary distribution in the kidney and tumor. The CDDP and PTX in Co-FA-NPs are mainly dispersed to the blood, liver, spleen, and tumor, followed by secondary distribution in the kidney and bone marrow. Compared with free-CDDP and free-PTX, co-delivery NPs have a greater tendency to distribute in the liver, spleen, bone marrow and tumor, mononuclear phagocyte system(MPS), and the circulating time in the blood was significantly longer than that of single free-CDDP and single free-PTX form. This conforms to the distribution characteristics of stealth NPs. Meantime, the blood drug concentration from stealth NPs was reduced slower and NPs can be gathered by EPR effect in tumor which is beneficial to tumor treatment. At the same time, the NPs produce a barrier effect between the chemotherapeutic drugs and the tissues of the body avoid direct contact. In addition, the slow release effect of NPs can significantly reduce the side effects of chemotherapeutic drugs. The CDDP and PTX in the co-delivery NPs have the same distribution and pharmacokinetic characteristics which is also beneficial for enhancement of the synergistic effects between the two kinds of chemotherapeutic drugs.

### *In vivo* antitumor efficiency

**Figure 8:**
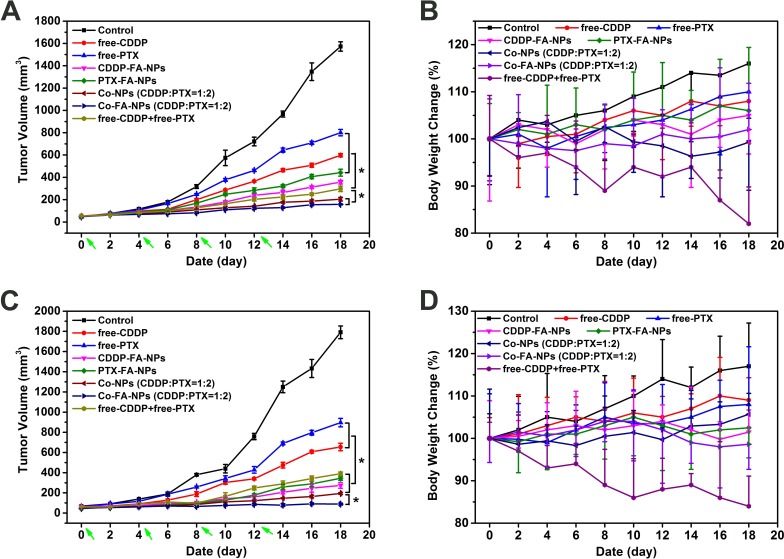
After PBS, free-CDDP, free-PTX, CDDP-NPs, PTX-NPs, Co-NPs and free-NPs + free-PTX treatment, tumor volume **A.** and body weight **B.** changes for xenograft A549 lung cancer; tumor volume **C.** and body weight **D.** changes for xenograft M109 lung cancer.

Based on the above results, the *in vivo* anticancer activity and systemic toxicity of the dual-drug loaded NPs were further investigated on M109 and A549 lung cancer cell grafted tumor-bearing nude mice. Mice were treated with PBS, free drug, drug-loaded NPs, and co-delivery NPs, respectively. The mice were injected every four days via intravenous injection, and tumor volumes and body weights were measured every two days. As shown in Figure 8, in comparison with the rapid tumor growth of PBS treatment group, all the drug formulations showed efficacy in inhibiting the tumor growth to different degrees. For the free drugs and the drug-loaded NPs treated groups, five conclusions could be summarized as follows: (1) the free-drug was effective for tumor-treatment. The combination of CDDP and PTX was more effective than the use of single drug. (2) In the A549 cells mice, for the same drugs, the loaded-drug NPs-treated mice showed less tumor volume compared with free-drug. The free drug combination treated mice have less tumor volume than single loaded-drug NPs-treated mice, and the difference was statistically significant. The best antitumor activity was observed in the Co-FA-NPs treated group which revealed the greatest inhibition of tumor growth. The tumor volume of Co-FA-NPs treated group was only 10.04% of control group at the end of experiment which was 3.78-fold, 5.07-fold, 1.29-fold and 1.87-fold smaller than that treated with free-CDDP, free-PTX, Co-NPs and free-CDDP + free-PTX, respectively.[2, 8, 10] In addition, there was no significant difference between Co-FA-NPs and Co-NPs treatment groups. (3) In the M109 cells mice, for the same drugs, the loaded-drug NPs-treated mice showed significantly less tumor volume in comparison to the free-drug treated mice (*p* < 0.05). The free drug combination treated mice have similar tumor volume when compared with single loaded-drug NPs-treated mice. The best antitumor activity was observed in the Co-FA-NPs treated group which exhibited almost complete inhibition of tumor growth and no obvious tumor recrudescence throughout treatment. The tumor volume of Co-FA-NPs treated group was only 4.97% of control group at the end of experiment, which was 7.36-fold, 10.08-fold and 4.38-fold smaller than that treated with free-CDDP, free-PTX, and free-CDDP + free-PTX, respectively. (4) In M109 cells grafted mice, the antitumor efficacy of Co-FA-NPs was better than that of Co-NPs treated grafted mice (*p* < 0.05). (5) According to the tumor relative growth rate (tumor volume of treated groups/control), the antitumor efficacy of Co-FA-NPs for M109 cells grafted mice was better than that in A549 cells grafted mice (*p* < 0.05). The superior antitumor effect of Co-FA-NPs could be attributed to the targeting, the enhanced nanoparticles stability during the blood circulation, the sufficient and coinstantaneous delivery of two drugs to the tumor site, the efficient cellular uptake in the tumor, and the synergistic effect of CDDP and PTX on tumor inhibition. [40]

Alteration of body weight is an indication of systemic toxicity. As depicted in Figure 8, in A549 cells grafted mice, the body weights of PBS, free-CDDP, free-PTX, CDDP-FA-NPs and PTX-FA-NPs treated mice showed a continuous and slow increase, which could be attributed to the tumor growth and low toxicity of chemotherapy at injection dosage. Obvious weight loss (about 15%) was observed in mice treated with free-CDDP and free-PTX co-administration. This enhanced toxicity of free drug combination limited the success of clinical combination chemotherapy. On the contrary, the treatment with co-delivery NPs did not lead to any significant body weight loss which reflects reduced systemic toxicity of the loaded drugs and no tumor growth. These effects have provided more options for enlargement of the therapeutic window. [41]

In M109 cells grafted mice, the body weights of PBS, free-CDDP, and free-PTX treated mice also showed a continuous and slow increase. In free-CDDP + free-PTX treated M109 cells grafted mice, similar weight loss was observed in comparison with A549 cells grafted mice. In addition, the treatment with CDDP-FA-NPs, PTX-FA-NPs and co-delivery NPs did not lead to any significant alteration of body weight.

The good *in vivo* performance (enhanced anti-tumor efficiency and lowered systemic toxicity) of the PTX + CDDP co-delivery NPs can be explained by the following reasons: (1) the inherent long circulation and EPR effect of stealth NPs would passively/actively accumulate the NPs at the tumor site; (2) the drugs cross-linking shell effectively protected the CDDP and PTX in the core domains against rapid clearance during blood circulation while quickly released the carriers in the tumor acidic condition; (3) the synergistic effect of the two drugs in the co-delivery NPs resulted in better tumor inhibition effect; (4) because of the sustained release behavior, the delivery system showed a continued tumor inhibition effect throughout the 12 day treatment period and the continued inhibition was observed until the mice were sacrificed following the last drug administration. [2, 8, 10] Based on the above results, with the high antitumor efficacy and the low drug-related toxicity, we conclude that the amphiphilic copolymer (FA/PEG-PLGA) co-delivery CDDP + PTX strategy provides a promising solution for cancer chemotherapy. For the tumor with folate receptor positive, Co-FA-NPs showed more surprising anti-tumor effects.

The principle of drug combination is to achieve efficient antitumor effect with minimal side-effects at lower drug doses. Therefore, our next goal is to reduce drug doses to obtain the maximal therapeutic effect and further reduce side effects.

### Histological analysis

**Figure 9:**
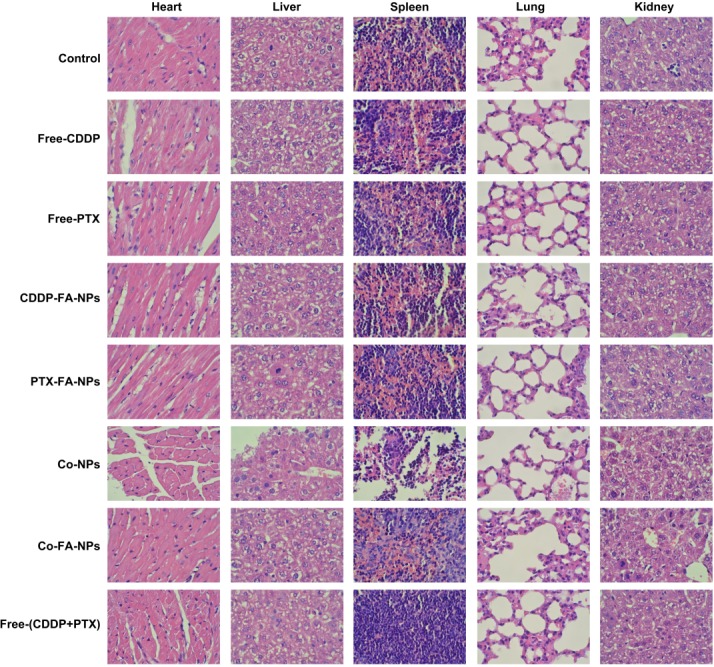
Histopathological analysis after 18-day tail vein administration of the various forms of drugs

To further investigate the side-effects of Co-FA-NPs, nude mice afflicted with tumors were euthanized and the heart, liver, spleen, lung and kidneys were dissected and stained with H&E for post-mortem analysis. The data of PBS, free-CDDP, free-PTX, CDDP-FA-NPs, PTX-FA-NPs, Co-NPs, Co-FA-NPs and free-CDDP + free-PTX treated groups are shown in Figure 9. For H&E staining, the normal cells were found to contain large nuclei with spherical or spindle shape and contained greater levels of chromatin. In comparison, the necrotic cells failed to maintain an adequate cell morphology and the chromatin changed and appeared to become smaller, pyknotic, or non-existent inside the cell. As shown in Figure 9, the organ cells with normal shape and more chromatin were observed in the PBS, CDDP-FA-NPs, PTX-FA-NPs, Co-NPs and Co-FA-NPs group and exhibited no obvious damage to organs which indicates few side-effects and a vigorous cell state. This is consistent with the body weight change result in Figure 8. However, the various degrees of liver/kidney tissue necrosis were observed in free-CDDP, free-PTX, and free-CDDP + free-PTX treated groups. Free-CDDP caused slight damage to the kidneys and free-PTX caused obvious damage to the liver. Free-PTX + free-CDDP caused severe damage to liver and kidneys. The free-CDDP + free-PTX treated group had large necrotic areas as compared with the groups treated with free-CDDP and free-PTX, which indicates that most liver and kidney cells were necrotic in the free-CDDP + free-PTX treated group. Together, this data clearly confirms that the level of necrosis and apoptosis, in liver and kidney tissue treated with co-delivery NPs, was significantly lower than that treated with free-CDDP + free-PTX. This, therefore, demonstrates that co-delivery NPs enhance the synergistic anti-tumor effect of CDDP and PTX in addition to reducing unwanted side effects.

### Effects on blood components after treatment with multiple drug formulations

The changes in blood components following treatment with PBS, free-CDDP, free-PTX, CDDP-FA-NPs, PTX-FA-NPs, Co-NPs, Co-FA-NPs and free-CDDP + free-PTX were shown in Table 4. The changes in blood components are primarily dependent on the administration of drug form. In treating the free-CDDP + free-PTX group, there were remarkable decreases observed for white blood cells (WBC) and neutrophils when compared with control (*p* < 0.05). In addition, there was an obvious increase in ALT, AST, serum creatinine, and BUN observed when compared with control (*p* < 0.05). These changes indicate that although the combination of free-CDDP + free-PTX has anti-tumor capabilities, they also have the potential to cause blood toxicity and liver and kidney function damage. The single free-CDDP and single free-PTX induces a slight increase of serum creatinine, BUN, ALT and AST, but did not differ significantly when compared with control. While the drug-loaded NPs remained constant when the formulations were given via injection through the tail vein, this indicates the NPs can enhance the anti-tumor effect of chemotherapeutic drugs. In addition, the NPs may decrease the toxicity and side-effects of chemotherapeutic drugs. Through the use of the co-delivery system, we can further enhance the synergistic effect of chemotherapeutic drugs and simultaneously decrease unwanted side-effects.

**Table 4:** Blood component changing following various drugs treatments

Groups	WBC (×10^9^/L)	Neutrophils (×10^9^/L)	Erythrocyte (×10^12^/L)	Plateletes (×10^9^/L)	ALT (U/L)	AST (U/L)	Scr (μmol/L)	BUN (mmol/L)
Control	5.81±1.47	1.15±0.26	10.14±0.87	498.78±78.64	28.26±8.46	20.71±2.23	132.62±19.87	6.81±2.11
Free-CDDP	5.57±2.13	0.98±0.14	10.53±1.77	463.27±91.43	31.17±11.32	22.63±1.44	167.42±21.36	8.24±3.45
Free-PTX	5.21±0.68	1.03±0.11	9.92±2.14	521.27±68.97	34.53±6.99	23.45±2.02	131.56±18.03	5.97±1.48
CDDP-FA-NPs	5.79±1.12	1.16±0.23	11.06±0.41	494.99±104.71	26.51±5.43	20.24±2.98	138.75±24.46	6.43±0.79
PTX-FA-NPs	5.64±1.89	1.08±0.09	9.87±0.78	502.41±64.32	30.11±13.71	21.12±1.47	123.33±15.23	7.44±2.87
Co-NPs (CDDP: PTX = 1:2)	5.51±1.32	1.05±0.18	10.02±2.14	540.65±89.39	34.87±6.99	22.95±3.26	153.72±16.33	6.94±1.31
Co-FA-NPs (CDDP: PTX = 1:2)	5.62±0.47	1.32±0.20	10.32±1.01	537.16±88.08	31.76±6.34	19.47±1.78	137.82±13.67	6.12±1.89
Free-CDDP+Free-PTX	4.26±1.43*	0.76±0.18*	9.89±0.93	401.3±141.73	44.92±10.49*	28.89±1.23*	226.54±31.24*	10.75±3.64*

## MATERIALS AND METHODS

### Materials

PEG with molecular weight (Mw) 2000 was purchased from Sigma-Aldrich (China) Co., Ltd. (Shanghai, China). CDDP, PTX, N-hydroxy-succinimide (NHS), and 1,3-diisopropylcarbodiimide (DIC) were obtained from Meloney Biotechnology Co., LTD (Dalian, China). Dialysis bags (MWCO 3500Da) and Cell Counting Kit-8 (CCK8) was purchased from Qcbio Science & Technologies Co., Ltd (Shanghai, China). L-lactide, Nε-carbobenzyloxy-L-lysine N-carboxyanhydride and glycolide were obtained from Bicoll Biotechnology (Shanghai) Co. Ltd. Bovine serum albumin (BSA) and FA was purchased from Mr. Ng Biological Technology Co., LTD (Nanjing, China). Pluronic 188 (F68) was obtained from BASF (Ludwigshafen, Germany). DMSO, ethyl acetate, etc, reagents and solvents were purchased from Sinopharm Chemical Reagent Co., Ltd (Shanghai, China). All cell culture media and reagents were obtained from Gibco (Grand Island, New York) unless otherwise specified.

Healthy male Balb-c/nude mice (aged 4 weeks) and male kunming mice (aged 6-8 weeks, approximately 20g) were obtained and reared in FuDan University. All animal studies were performed as the “Guide for the Care and Use of Laboratory Animals”.

L929 (rat fibroblasts), R 1610 (hamster lung cell), M109 with folic acid receptor (FR) positive (murine lung carcinoma cell) and FR-negative A549 (human lung cancer cell) cells were grown in high-glucose DMEM medium containing 10% fetal bovine serum (FBS), 100 *μ*g/mL streptomycin and 100 U/mL penicillin at 37°C under 5% CO_2_. [11, 12, 13, 14]

### Synthesis of the FA-PEG-PLGA polymer

The synthesis of FA-PEG-PLGA polymer (Mw 12,000) was, as with the previous literatures, with minor modifications. [15, 16, 17] In brief; (1) the hydroxyl-terminated PEG-PLGA was synthesized; (2) the hydroxyl end-group converted to Boc-_L_-Phe; (3) the t-Butoxycarbonyl end-group was removed and synthesis amino-terminated PEG-PLGA; (4) amino-terminated PEG-PLGA (200 *μ*mol) was dissolved in DMSO (60 mL), then mixed with NHS (1,000 *μ*mol), DIC (1,000 *μ*mol) and folic acid (500 *μ*mol) at 37°C. After 24 hours, the solution was mixed with 200 mL of distilled water and then centrifuged at 2,500×g. After discarding the sediment, the supernatant was dialyzed and freeze-dried. The concentration of conjugated folic acid was determined at 365 nm (UV absorbance value) after the obtained product was dissolved in DMSO. [16] Different concentrations of folic acid in DMSO were used as reference.

### Preparation of the FA-PEG-PLGA NPs (blank NPs), CDDP-FA-PEG-PLGA NPs (CDDP-FA-NPs), PTX-FA-PEG-PLGA NPs (PTX-FA-NPs), Co-FA-NPs, and CDDP-PTX-PEG-PLGA NPs (Co-NPs)

The blank NPs and CDDP-FA-NPs were prepared by the double emulsion (W/O/W) method with a little modification as described by Wang, et al. [18] In brief, the CDDP solution (1.0 mg in 100 *μ*L solution consisting of water and DMSO (3/2, v/v)) or 100 *μ*L of distilled water was emulsified in FA-PEG-PLGA solution (20 mg in 1,000 *μ*L of ethyl acetate by sonication (400 W, 10 s × 6 times) (JY 92-II ultrasonic processor; Ningbo Scientz Biotechnology Co., Ltd., China). Subsequently, the emulsion was added to 10.0 mL of F68 solution (1%, w/w) and sonicated again (400 W, 10 s × 8 times). After being rotated and evaporated, the solution was centrifuged for 30 min at 15,000 rpm under 4°C. Finally, the collected NPs were washed twice using distilled water and freeze-dried. [2, 10]

PTX-FA-NPs were prepared using an emulsion/solvent evaporation method. A solution of 5 mg of FA-PEG-PLGA and 0.25 mg of PTX was dissolved in 0.25 mL of ethyl acetate. Then, the mixture was added into 10.0 mL of F68 solution (1%, w/w), and emulsified by sonication. The subsequent steps were identical to the preparation of CDDP-FA-NPs.

Co-NPs and Co-FA-NPs was produced by the improved double emulsion technique. In brief, copolymer (20 mg) and PTX (0.5, 1 or 2 mg) dissolved in 1 mL of ethyl acetate, mixed with 0.1 mL of CDDP solution (10 mg/mL). The mixture was then sonicated for 1 min. Then, 50 *μ*L of emulsion was added into 3.9 mL of stirring F68 solution (1%, w/w), and once again emulsified by sonication. The subsequent steps were the same as the preparation of CDDP-FA-NPs.

### Physical characterization of NPs

The size and zeta potential of the NPs were characterized using a ZetaSizer Nano ZS (Malvern Instruments Ltd., UK). The size profiles of the NPs were determined by dynamic light scattering (DLS). The zeta potential (ζ) was determined through a He-Ne laser beam with a wavelength of 633.8 nm at 25°C. Three batches of samples were analyzed. The morphology of NPs was observed with a JEM-1400 transmission electron microscope (TEM) (JEOL, Japan). [19, 20]

### Stability study and *in vitro* drug release assay

The phosphate-buffered saline (PBS) (0.1 M, pH 5.0, 7.4 or 9.1), 5% FBS and 2% BSA solution with 100 *μ*g/mL streptomycin and 100 U/mL penicillin were prepared and filtered.

A total of 5 mg of NPs were suspended in 15 mL of 5% FBS, 2% BSA or PBS with continuous stirring under 128 rpm at 37°C. The sizes of NPs were detected at 0, 1, 2, 3, and 5 days in order to determine the stability of the NPs by DLS. [21, 22]

For *in vitro* drugs release assay, 20 mg of CDDP-FA-NPs, PTX-FA-NPs, Co-FA-NPs, or Co-NPs were re-suspended in 8 mL of 5% FBS, 2% BSA or PBS containing F68 (1%, w/w) and transferred to dialysis bags. [23, 24] Then, the NPs were placed in 60 mL of 5% FBS, 2% BSA or PBS containing F68 (1%, w/w) with continuous stirring under 128 rpm at 37°C. At a predetermined time point, the medium was replaced with fresh medium. The taken medium were mixed with methanol to precipitate the unneeded protein, centrifuged, and the supernatant was assayed using a high performance liquid chromatography (HPLC) to determine the concentration of the released CDDP and PTX in the medium. The accumulative percentage of the released CDDP and PTX was determined according to the calibration curve. Serially diluted concentrations of CDDP and PTX standard were used to construct the calibration curve. An Agilent 1200 series UV detector and chemstation system were used at 25°C.

CDDP was determined using an Eclipse XDB-C18 column (150 × 4.6 mm, 5 *μ*m) at 25°C. The flow rate of the mobile phase (methanol: 0.9% sodium chloride solution = 80: 20, v/v) was 1 mL/min and the UV detector wavelength was 310 nm. A sample volume of 20 *μ*L was injected into HPLC column for each analysis.

For PTX, the mobile phase was water/acetonitrile (1:1, v/v) and the test wavelength was 227 nm. The other conditions were identical with the detection of CDDP.

Drug loading efficiency (DLE, wt%) of the NPs was determined as the equation:

DLE=(weight of loaded drug/total weight of drug)×100%.

Drug loading content (DLC, wt%) of the NPs was determined as the equation: DLC = (weight of loaded drug/weight of drug-loaded NPs) × 100%.

### Cytotoxicity *in vitro*

Cells were cultured in 96-well plates with 100 *μ*L of DMEM medium (10% FBS, 100 *μ*g/mL streptomycin, and 100 U/mL penicillin) in the incubator until the cells adhered. Then, the medium was replaced with 200 *μ*L of DMEM medium containing blank NPs (equal to the FA-PEG-PLGA concentration in the Co-FA-NPs (CDDP:PTX = 1:1)), free-CDDP, free-PTX, CDDP-FA-NPs, PTX-FA-NPs, Co-NPs, Co-FA-NPs or free-CDDP + free-PTX. DMEM medium was used as control. The total drug content, in all the test groups, was constant at 10.0 *μ*g/mL. After 24 hours, the relative growth rate (RGR) of cells was determined via a CCK-8 assay as instruction. [25, 26]

### Pharmacokinetic analysis

Before the pharmacokinetic experiment, the kunming mice were fasted for at least 8 hours and water was given *ad libitum*. These mice were divided into a number of experimental groups at random. Group I received free-CDDP (5 mg/kg), Group II received free-PTX (12 mg/kg), Group III received CDDP-FA-NPs (CDDP equivalent to 5 mg/kg), Group IV received PTX-FA-NPs (PTX equivalent to 12 mg/kg), and Group V received Co-FA-NPs (equivalent to CDDP of 5mg/kg and PTX of 12 mg/kg) via tail vein. At predetermined time intervals (5, 15, 30 min and 1, 3, 5, 10, and 24 h), mice were sacrificed via cardiac puncture under diethyl ether anesthesia. A sample of 0.2 mL of blood was collected into tubes containing heparin and centrifuged for 15 min at 2,500×g under 4°C to obtain plasma. The pharmacokinetic parameters which terminal half-life (t_1/2_), area under the curve from time 0 h to 24 h (AUC_24h_), mean residence time (MRT), total body clearance (CL), and volume of distribution at steady state (V_d, ss_) were calculated by non-compartmental analysis using WinNonlin^TM^ (Pharsight Corporation, USA). The program of Pharmacological Calculation System and Duncan's multiple range tests was used to analyze the statistical significance. [27, 28]

### *In vivo* biodistribution study and antitumor activity

The A549 and M109 cells (1×10^6^ cells, 0.1 mL in PBS) were subcutaneously transplanted into the right flank of male Balb-c/nude mice, respectively. When the tumor volume was approximately 50-70 mm^3^ and 30-50 mm^3^, the biodistribution study and treatment was initiated, respectively (*n* = 6).

In the biodistribution study, M109 lung cancer cell grafted, tumor-bearing, nude mice were injected free-CDDP (5 mg/kg), free-PTX (12 mg/kg), Co-NPs (CDDP: PTX = 1:2) or Co-FA-NPs (CDDP: PTX = 1:2) via tail vein. At predetermined time intervals (5, 15, 30 min and 1, 3, 5, 10, and 24 h), mice were sacrificed via cardiac puncture under diethyl ether anesthesia. Liver, spleen, muscle, bone marrow, kidney, small intestine, blood, and tumor were collected, washed with PBS, and weighed. The plasma and homogenized organs were mixed with methanol to precipitate the unneeded protein, centrifuged, and the supernatant was detected by HPLC analysis.

In the antitumor activity experiment, mice were treated with PBS, free-PTX (1.2 mg/kg), free-CDDP (0.5 mg/kg), CDDP-FA-NPs (equivalent to 0.5 mg CDDP/kg), PTX-FA-NPs (equivalent to 1.2 mg PTX/kg), Co-FA-NPs (CDDP: PTX = 1:2, DLC equivalent to CDDP of 0.5mg/kg and PTX of 1.2 mg/kg), Co-NPs (CDDP: PTX = 1:2, DLC closely equivalent to CDDP of 0.5mg/kg and PTX of 1.2 mg/kg, the total drugs were 1.7 mg/kg) and free-CDDP + free-PTX (equivalent to 0.5 mg CDDP/kg and 1.2 mg PTX/kg) intravenously via tail vein on days 0, 4, 8, and 12. The tumor volume and the body weight were evaluated every two days to determine the antitumor activities and the side effects. The estimated tumor volume (mm^3^) was calculated according to the formulations: V = *a·b*^2^/2 (*a* and *b* was the longest and shortest diameter, respectively). At day 18, mice were sacrificed. [29] Approximately 200 *μ*L of blood was collected with microtubes (pretreated with heparin) to detect the blood component change and to evaluate for any potential systematic toxicology.

### Hematotoxicity tests and Hisopathological examination

On day 18, mice were anesthetized and the chests were excised open. To evaluate for any potential systematic toxicology, approximately 20 *μ*L of blood was collected from the heart via the use of the plastic syringe that was pre-treated with15 mM EDTA. The number of blood cells present in whole blood was calculated by the using an Auto-analyzer (XT-1800i, Sysmex Corporation, Japan). A second blood sample (around 150 *μ*L) was collected from the heart via the use of a plastic syringe that was pre-treated with heparin sodium (10 IU). Plasma was obtained by centrifuging the blood sample at 3,000 rpm for 20 min. The concentrations of aspartate aminotransferase (AST), alanine aminotransferase (ALT), serum creatinine (Scr), and blood urea nitrogen (BUN) were calculated with the use of a Biochemical Auto-analyzer (7170S, Hitachi, Japan). [30, 31]

The heart, liver, spleen, lung, and kidney were collected, fixed immediately in 4% buffered paraformaldehyde for 12 hours, and embedded in paraffin block. Then, the paraffin block was sliced at 5*μ*m thickness using a Leitz model 1512 microtome. Then, the 5*μ*m paraffin slices were stained with hematoxylin-eosin (H&E). Finally, these finished slices were observed using a light microscope and the representative graphs were saved.

### Statistical analysis

All analyses were compiled using SPSS 20.0 software. The results were presented as the mean ± standard deviation (SD) for the resultant values that were obtained from a minimum of three independent experiments. Statistical analysis was performed by incorporating the use of a one-way analysis of variance (ANOVA) and Tukey's post hoc test. All tests are accepted as statistically significant when the *p* value is less than 0.05.

## CONCLUSIONS

We present an FA modified amphiphilic copolymer-based CDDP and PTX combination strategy for greater chemotherapeutic response and lessened side-effects. Core-shell-corona particles were obtained by entrapping CDDP and PTX in the FA-PEG-PLGA diblock copolymer. The resultant dual-drug-loaded particles showed to work in synergy to promote the inhibition of the proliferation of A549 and M109 lung cancer cells. An *in vivo* study showed that the FA modified amphiphilic copolymer-based combination of PTX and CDDP displayed greater inhibition toward A549 and M109 tumor growth than free drug combinations, with less unpleasant side effects. In addition, FA modified amphiphilic copolymer-based combination of PTX and CDDP showed greater anti-tumor effects for M109 tumor with folic acid receptor positive than that for A549 tumor with folic acid receptor negative. Therefore, this system could be highly beneficial for cancer chemotherapy. In particular, this drug combination could be especially useful for treatment of tumors with folate receptor positive.

## SUPPLEMENTARY MATERIAL SCHEME


