# Supplementary material for: Co-delivery of cisplatin and paclitaxel by folic acid conjugated amphiphilic PEG-PLGA copolymer nanoparticles for the treatment of non-small lung cancer
Source: Oncotarget. 2015 Oct 26;6(39):42150–68. doi: 10.18632/oncotarget.6243 (PMC4747216; doi:10.18632/oncotarget.6243)
Supplement: Supplementary file 1 [file oncotarget-06-42150-s001.pdf]

Co-delivery of cisplatin and paclitaxel by folic acid conjugated amphiphilic PEG-PLGA copolymer nanoparticles for the treatment of non-small lung cancer

Supplementary Material

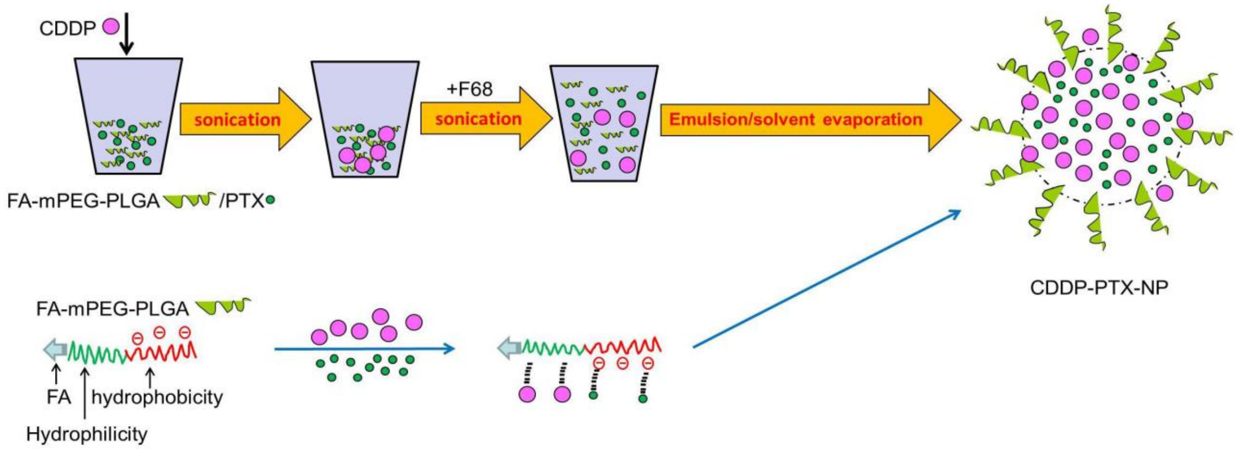

Supplementary **Scheme 1.** Schematic illustrations of antitumor drug loading.
